# Electrodeposition of Manganese-Nickel Oxide Films on a Graphite Sheet for Electrochemical Capacitor Applications

**DOI:** 10.3390/ma7010265

**Published:** 2014-01-09

**Authors:** Hae-Min Lee, Kangtaek Lee, Chang-Koo Kim

**Affiliations:** 1 Department of Chemical Engineering and Division of Energy Systems Research, Ajou University, Suwon 443-749, Korea; E-Mail: yihaemin@gmail.com; 2 Department of Chemical and Biomolecular Engineering, Yonsei University, Seoul 120-749, Korea; E-Mail: ktlee@yonsei.ac.kr

**Keywords:** Mn-Ni oxide films, electrodeposition, supercapacitor, specific capacitance, cycling stability

## Abstract

Manganese-nickel (Mn-Ni) oxide films were electrodeposited on a graphite sheet in a bath consisting of manganese acetate and nickel chloride, and the structural, morphological, and electrochemical properties of these films were investigated. The electrodeposited Mn-Ni oxide films had porous structures covered with nanofibers. The X-ray diffractometer pattern revealed the presence of separate manganese oxide (γ-MnO_2_) and nickel oxide (NiO) in the films. The electrodeposited Mn-Ni oxide electrode exhibited a specific capacitance of 424 F/g in Na_2_SO_4_ electrolyte. This electrode maintained 86% of its initial specific capacitance over 2000 cycles of the charge-discharge operation, showing good cycling stability.

## 1. Introduction

Electrochemical capacitors are attracting significant interest owing to their potential role in power storage of electric and fuel cell vehicles. Electrochemical capacitors can be classified into two types according to their charge storage mechanisms [1,2]: electric double layer capacitor (EDLC) and redox supercapacitor (or pseudo-capacitor). EDLCs mainly utilize the nonfaradaic charge separation at the electrode/electrolyte interface. Redox supercapacitors make use of a reversible redox reaction in order to store charges. The behavior of redox supercapacitors is typically termed “pseudo-capacitance” and resembles a rechargeable battery more than a traditional capacitor. In the case of redox supercapacitors, various noble and transition metal oxides (e.g., RuO_2_, CoO*_x_*, NiO*_x_*, MnO_2_, *etc.*) and conducting polymers have been employed as electrode materials [3–7]. Among these materials, ruthenium oxide has been found to be a successful redox supercapacitor exhibiting a specific capacitance up to 720 F/g [3]. However, cost is a prohibitive factor in the large-scale commercial production of ruthenium oxide as a redox supercapacitor.

Manganese and its oxides have received much attention as an alternative to the ruthenium oxide in electrochemical capacitor applications because of their satisfactory electrochemical performance, relatively low cost, natural abundance, and so on [5,8,9]. Several methods, including hydrothermal synthesis [10], chemical bath deposition [11], sol-gel method [12], and co-precipitation [13], have been used to produce manganese oxide electrodes for electrochemical capacitors. However, these methods produce oxide powders and the use of oxide powders is complicated and inconvenient in making electrodes. It needs several sequential treatments such as synthesizing powders, mixing them with a conducting agent and a binder, and then making electrodes [14]. A more effective method to prepare manganese oxide electrodes for electrochemical capacitor applications is electrodeposition because it leads to a direct deposition of oxide films on an electrode. In addition, electrochemical manganese dioxides which are in the forms of α-, β-, and γ-MnO_2_ exhibit better electrochemical properties for electrochemical capacitor applications than natural manganese dioxide and chemically prepared manganese dioxide [15]. There are also several reports that binary metal oxides such as manganese-nickel oxide and manganese-cobalt oxide enhance the electrochemical capacitive performance of manganese oxides and have better electrochemical properties than single metal oxide [16–20].

In the present work, we have developed a bath consisting of manganese acetate and nickel chloride for the electrodepositon of manganese-nickel (Mn-Ni) oxide films as electrochemical capacitor electrodes. The structural, morphological, and electrochemical properties of the electrodeposited manganese-nickel oxide films were discussed.

## 2. Results and Discussion

### 2.1. Formation Mechanism of the Electrodeposited Mn-Ni Oxide Films

The anodic electrodeposition of manganese oxide in an electrolyte containing Mn^2+^ ions can be described by the following Equation [9]:

$${\mathrm{Mn}}^{2+}+2H_{2}O\to {\mathrm{MnO}}_{2}+4H^{+}+2e^{-}$$ (1)

In Equation (1), the oxidation state of Mn increases from +2 to +4. Since it is improbable that two electrons are transferred in a single step, the formation of MnO_2_ from Mn^2+^ occurs concomitantly with the oxidation of water molecules. The detailed intermediate steps for the overall reaction of Equation (2) are as follows:

$${\mathrm{2Mn}}^{\text{2}+}\to {\mathrm{2Mn}}^{\text{3}+}+{\mathrm{2e}}^{-}$$ (2)

$${\mathrm{2Mn}}^{\text{3}+}\to {\mathrm{Mn}}^{\text{2}+}+{\mathrm{Mn}}^{\text{4}+}$$ (3)

$${\mathrm{Mn}}^{\text{4}+}+{\mathrm{4H}}_{\text{2}}\text{O }\to \mathrm{Mn}{\left(\mathrm{OH}\right)}_{\text{4}}+{\mathrm{4H}}^{+}$$ (4)

$$\mathrm{Mn}{\left(\mathrm{OH}\right)}_{\text{4}}\to {\mathrm{MnO}}_{\text{2}}+{\mathrm{2H}}_{\text{2}}O$$ (5)

The deposition of nickel oxide makes a use of nickel hydroxide precipitation. Nickel hydroxide is formed on the electrode such that:

$${\text{Ni}}^{\text{2}+}+{\text{ 2OH}}^{-}\to \text{ Ni}{\left(\text{OH}\right)}_{\text{2}}$$ (6)

Nickel hydroxide is subsequently involved in the electrochemical reactions of Equations (8) and (9) [21,22]:

$$\text{Ni}{\left(\text{OH}\right)}_{\text{2}}+{\text{ OH}}^{-}\to \text{ NiO}-\text{OH }+{\text{ H}}_{\text{2}}\text{O }+{\text{ e}}^{-}$$ (7)

$$\text{NiO}-\text{OH }+{\text{ OH}}^{-}\to {\text{ NiO}}_{\text{2}}+{\text{ H}}_{\text{2}}\text{O }+{\text{ e}}^{-}$$ (8)

Finally, the eletrodeposition of nickel oxide in an electrolyte containing Ni^2+^ ions can be represented by the Equation (10):

$$\text{Ni}{\left(\text{OH}\right)}_{2}+\;2{\text{OH}}^{-}\to {\text{ NiO}}_{2}+\;2H_{\text{2}}\text{O }+\;2e^{-}$$ (9)

### 2.2. Structural, Morphological, and Electrochemical Properties of the Electrodeposited Mn-Ni Oxide Films

**Figure 1.**
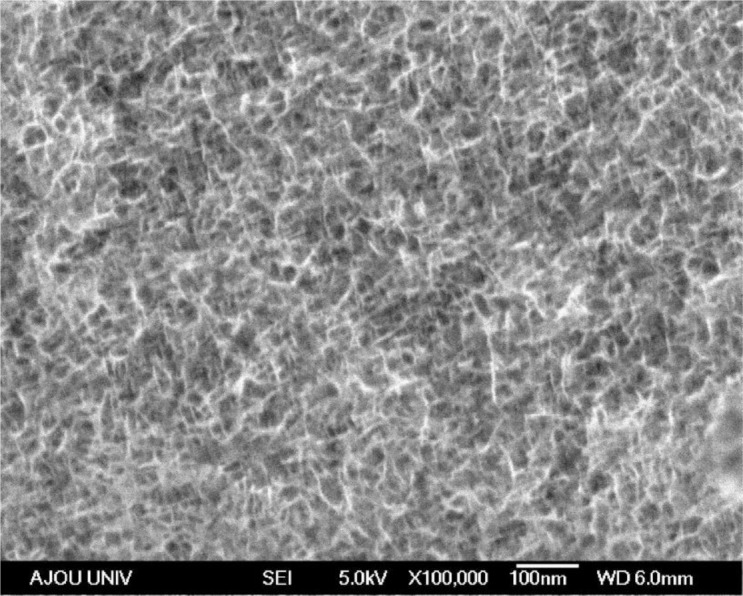
SEM image of the electrodeposited Mn-Ni oxide film.

The SEM (Hitachi, Tokyo, Japan) image of the electrodeposited Mn-Ni oxide film is shown in Figure 1. The substrate was well covered with nanofibers. Porous spaces were observed between the nanofibers, enhancing the redox process. This type of morphology is favorable for electrochemical capacitor applications owing to its large surface area.

**Figure 2.**
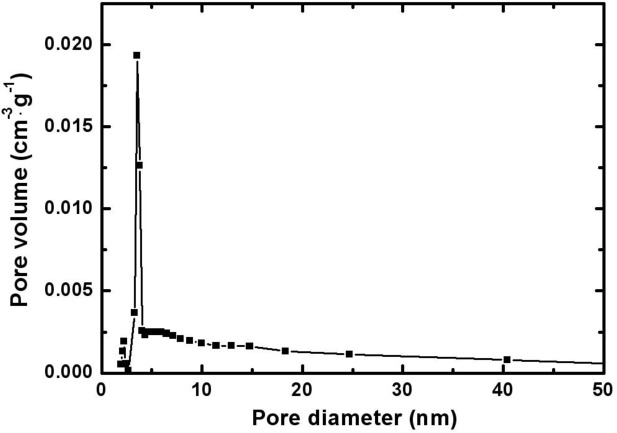
Pore size distribution of the electrodeposited Mn-Ni oxide film.

Figure 2 represents the pore size distribution of the electrodeposited Mn-Ni oxide film. The size of the pores in the film was mainly distributed in the range of 2–20 nm.

**Figure 3.**
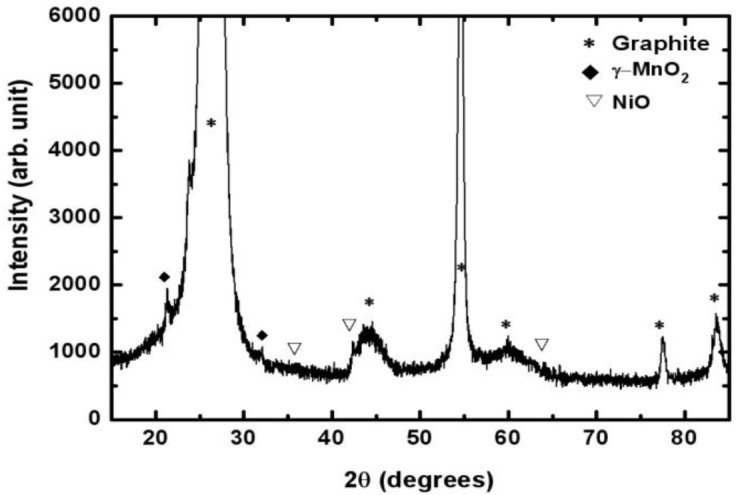
XRD spectrum of the electrodeposited Mn-Ni oxide film.

Figure 3 shows the XRD (Rigaku, Tokyo, Japan) patterns of the Mn-Ni oxides electrodeposited on a graphite sheet. The peaks at 26.4°, 44.4°, 54.5°, 59.7°, 77.2°, and 83.2° represented carbon in the substrate (graphite sheet) [JCPDS No. 41-1487]. The peaks at 22.1° and 34.4° indicated that manganese oxide existed in the form of γ-MnO_2_ [JCPDS No. 14-0644]. Nickel oxides were found to be in the form of NiO, resulting from the peaks at 37.2°, 43.2°, and 62.8°. The XRD spectrum suggested that separate manganese oxide (γ-MnO_2_) and nickel oxide (NiO), not in the form of composites, were obtained using electrodeposition.

**Figure 4.**
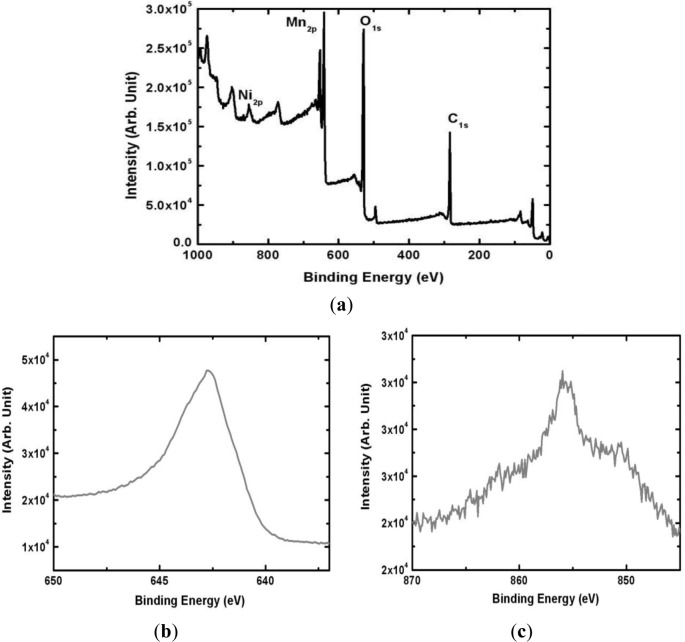
XPS spectra of the electrodeposited Mn-Ni oxide films. (**a**) survey scan; (**b**) Mn 2P_3/2_ electron; and (**c**) Ni 2P_3/2_ electron.

The surface composition of the electrodeposited Mn-Ni oxide film was analyzed by XPS (Thermo Fisher Scientific, East Grinstead, UK). Figure 4 shows the XPS spectrum of the Mn-Ni oxide film electrodeposited from an electrolyte containing 0.1 M of manganese acetate and 0.2 M of nickel chloride. The survey scan (Figure 4a) shows that the film contained Mn, Ni, and O. The peak representing C due to the substrate (graphite sheet) was also observed. A higher resolution of the Mn 2P_3/2_ and Ni 2P_3/2_ spectra revealed that the peak at 642.6 eV corresponded to manganese (IV) oxide [23] while the peak at 855.2 eV represented the presence of nickel (II) oxide [24] in the film. A quantitative analysis using XPS yielded a surface composition of Mn_0.24_Ni_0.05_O_0.71_.

**Figure 5.**
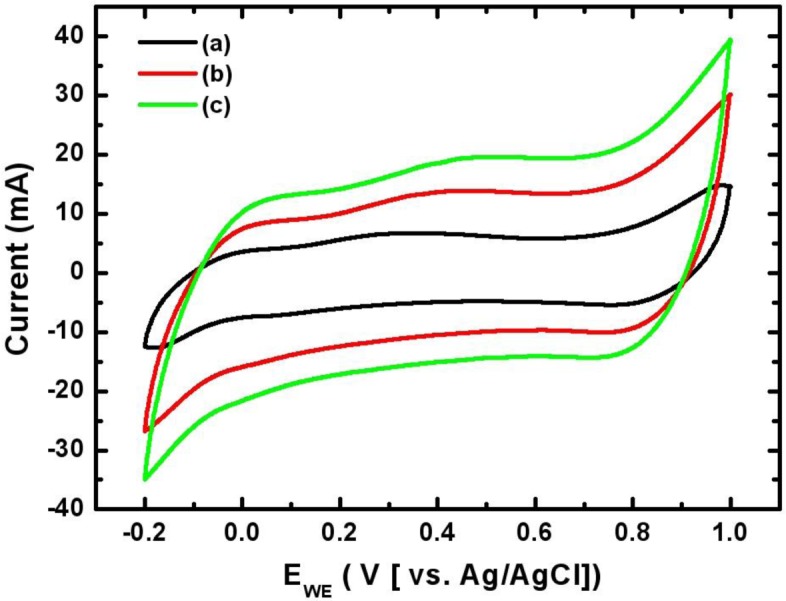
Cyclic voltammograms of the electrodeposited Mn-Ni oxide electrode in 0.5 M Na_2_SO_4_ at scan rates of (**a**) 20; (**b**) 60; and (**c**) 100 mV/s.

Figure 5 shows the cyclic voltammograms of the Mn-Ni oxide electrode electrodeposited at various scan rates. CVs were measured from −0.2 to +1.0 V *vs*. Ag/AgCl in the 0.5 M Na_2_SO_4_ electrolyte. The CV curve was shaped as an asymmetrically closed box at all scan rates. This indicates that the capacitance characteristics of the electrodeposited Mn-Ni oxide electrode are distinguished from the electric double-layer capacitance whose CV curve is nearly an ideal rectangular shape. Figure 5 also shows that the area under the curve increased with the scan rate. This implies that the voltammetric current was proportional to the scan rate, corresponding to a capacitive behavior. Based on the CV measurements, the capacitance of the electrodeposited Mn-Ni oxide electrode was calculated from Equation (10) in “Experimental Section”. The specific capacitance of the electrodeposited Mn-Ni oxide electrode was found to be 424 F/g at a scan rate of 20 mV/s in 0.5 M Na_2_SO_4_ electrolyte.

**Figure 6.**
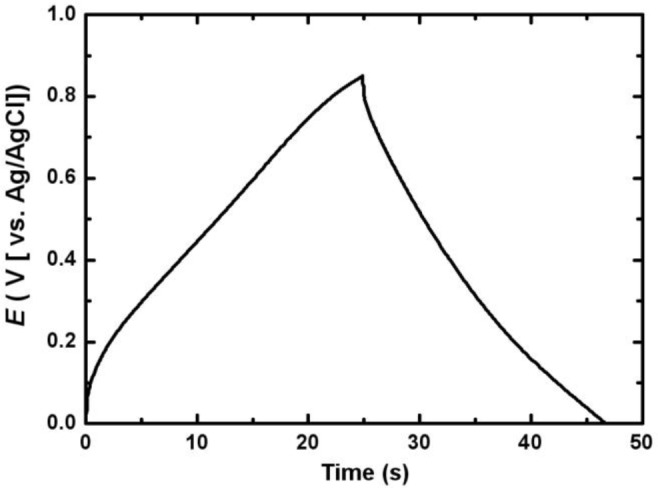
Charge-discharge curve of the electrodeposited Mn-Ni oxide electrode in 0.5 M Na_2_SO_4_.

Figure 6 shows the first charge-discharge cycle of the electrodeposited Mn-Ni oxide electrode. The charge-discharge curve was nearly symmetric in the range between 0 and 0.85 V (*vs*. Ag/AgCl), indicating that a good capacitive behavior of the electrodeposited Mn-Ni oxide electrode was observed.

**Figure 7.**
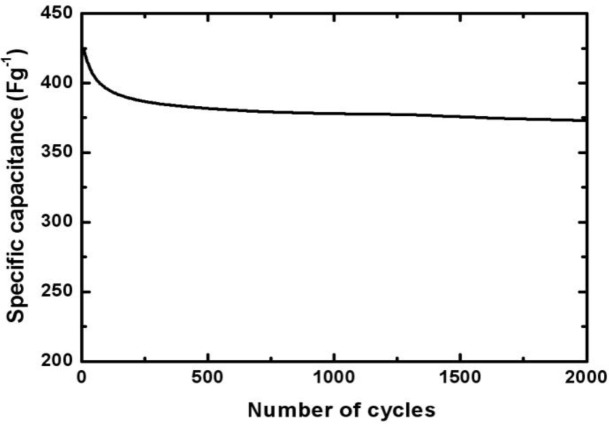
Specific capacitance of the electrodeposited Mn-Ni oxide electrode 0.5 M Na_2_SO_4_.

To evaluate the stability of the Mn-Ni oxide electrode, the charge-discharge cycling test at a current of 5 mA test was performed. Figure 7 shows the capacitance of the Mn-Ni oxide electrode over 2000 cycles. The specific capacitance decreased to about 380 F/g (90% of the initial value) during the initial 200 cycles. Then, it decreased slightly and became stable during the next 1800 cycles. The capacitance was about 370 F/g for 2000 cycles. This indicated that after 2000 cycles of the charge-discharge operation, the electrodeposited Mn-Ni oxide maintained 86% of its initial specific capacitance and exhibited good cycling stability.

## 3. Experimental Section

Mn-Ni oxide films were electrodeposited on a graphite sheet. The graphite sheet was cut into a 10 × 50 mm^2^ rectangular substrate and lacquered to expose an area of 10 × 20 mm^2^ for electrodeposition. Prior to electrodeposition, the substrate was polished with a silicon carbide paper. The polished substrate was etched in a 20 wt% sulfuric acid solution at room temperature for 20 s, and dipped into a solution of 2 M methanol for 20 s to remove macro-level surface defects and contamination. Then, the substrate was cleaned in double distilled water and dried in an oven at 65 °C for 6 h.

Manganese acetate (Mn(CH_3_COO)_2_ 4H_2_O) and nickel chloride (NiCl_2_ 6H_2_O) were used as the sources of manganese and nickel, respectively. The concentrations of manganese acetate and nickel chloride were 0.1 M and 0.2 M, respectively. The electrolytes were obtained by dissolving the above chemicals in deionized water. All chemicals were of analytical reagent grade supplied by Sigma-Aldrich (St. Louis, MO, USA).

The electrodeposition was carried out using a standard three-electrode cell. A saturated Ag/AgCl electrode was used as a reference electrode and a graphite sheet having a size of 20 × 70 mm^2^ (exposed area of 20 × 35 mm^2^) prepared by the same aforementioned method was used as a counter electrode. During electrodeposition, a constant potential of 1.2 V versus the reference electrode of Ag/AgCl from a stagnant electrolyte at room temperature (24 ± 1 °C) was applied using a computer-controlled potentiostat (Princeton Applied Research, VSP). The electrodeposition was done for 60 s in all cases. After deposition, the samples were washed with a deionized water jet, dried with flowing nitrogen gas, and annealed in an oven at 250 ± 1 °C for 3 h. Annealing of the samples was done to improve the adhesion of the Mn-Ni oxide films to the substrate.

The weight of the electrodeposited manganese-nickel oxides films were determined by a microbalance to an accuracy of 10 μg. The surface composition of the film was identified using X-ray photoelectron spectroscopy (XPS). Microstructural analysis of the film was carried out using a high-power X-ray diffractometer (XRD, Rigaku, D/max- 2500V/PC), which used a Cu K_α_ radiation (wavelength = 0.154 nm) as an incident beam and worked at 40 kV and 150 mA. Surface morphology of the film was examined using a field emission scanning electron microscopy (FE-SEM, Hitachi, S-4800, Tokyo, Japan). Nitrogen adsorption-desorption measurements were made using an adsorption analyzer (Micrometrics, ASAP 2010, Norcross, GA, USA) to obtain the pore size of the electrodeposited Mn-Ni oxide film. The sample was outgassed for 6 h at 473 K prior to the measurements. The pore sizes were calculated based on the Barret-Joyner-Halenda (BJH) desorption method.

For electrochemical measurements, a platinum-coated titanium mesh (2.5 cm^2^ in size) and a saturated Ag/AgCl electrode were used as a counter electrode and a reference electrode, respectively. Cyclic voltammograms (CVs) were recorded between −0.2 and 1.0 V versus Ag/AgCl. The capacitance (*C*) of an electrode can be calculated from the following Equation

$$C=\frac{dQ}{dV}=\frac{I}{dV/dt}$$ (10)

where *Q* is the charge on the electrode, *V* is its potential, *I* is the average current, and d*V*/d*t* is the voltage scanning rate. The specific capacitance of the electrodeposited manganese-nickel oxide electrode was obtained by dividing the capacitance by its respective weight. The weight of the electrodeposited manganese-nickel oxide films was measured using a microbalance with an accuracy of 10 μg. The constant current charge-discharge behavior of the manganese-nickel oxide electrode was investigated galvanostatically at a current of 5 mA.

## 4. Conclusions

The electrodeposition of Mn-Ni oxide films for use as an electrochemical capacitor electrode was carried out in a bath containing manganese acetate and nickel chloride. SEM studies showed that the Mn-Ni oxide film was well covered with numerous nanofibers in a three-dimensional network. Porous spaces were also observed between the nanofibers, which are required for electrochemical capacitor applications. The XRD analysis revealed that the electrodeposited Mn-Ni oxide film contained separate manganese oxide (γ-MnO_2_) and nickel oxide (NiO). The XPS spectra of the electrodeposited Mn-Ni oxide film confirmed the presence of manganese (IV) oxide and nickel (II) oxide. From the XPS measurements, the surface composition of the film was determined to be Mn_0.24_Ni_0.05_O_0.71_. The cyclic voltammetry measurements showed that the electrodeposited Mn-Ni oxide electrode had a specific capacitance of 424 F/g at a scan rate of 20 mV/s. The stability test of the film exhibited good cycling stability in that the electrodeposited Mn-Ni oxide electrode maintained 86% of its initial specific capacitance over 2000 cycles of the charge-discharge operation. Therefore, electrodeposition is a simple and promising method to fabricate Mn-Ni oxide films for electrochemical capacitor applications.
